# Cannabinoids for pain management in rheumatoid arthritis: a scoping review of clinical evidence and mechanisms

**DOI:** 10.1007/s00296-026-06314-x

**Published:** 2026-10-05

**Authors:** Nikolaos Zintziovas, Agathi Karakosta, Paraskevi Voulgari, Georgios Stefanakis, Eleni Arnaoutoglou, Petros Tzimas, Georgia Micha

**Affiliations:** 1 Faculty of Medicine, Department of Rheumatology Ioannina, University of Ioannina, Ipiros, Greece; 2 Faculty of Medicine, Department of Anesthesiology Ioannina, University of Ioannina, Ipiros, Greece; 3 Faculty of Medicine, Department of Anesthesiology Larissa, University of Thessaly, Thessalia, Greece; 4 Department of Rheumatology, University Hospital of Ioannina, Stavrou Niarchou Street, Ioannina, 45500 Greece

**Keywords:** Arthritis, Rheumatoid, Cannabinoids, Chronic pain, Pain management, Pain perception

## Abstract

Persistent pain remains a major unmet need in rheumatoid arthritis (RA), even in patients with adequately controlled inflammatory disease. Cannabinoids have been proposed as potential modulators of pain and inflammation, but their clinical role in RA remains uncertain. To map and critically appraise the available clinical evidence on cannabinoid-related interventions or exposures for pain management in adults with RA. PubMed/MEDLINE, Scopus, the Directory of Open Access Journals, and the Cochrane Central Register of Controlled Trials were searched for studies published from January 1, 1990, to September 10, 2026. Randomized controlled trials and observational studies evaluating cannabinoid-related interventions or exposures in adults with confirmed RA and reporting pain-related outcomes were eligible. Risk of bias was assessed using Cochrane RoB 2 for randomized trials and ROBINS-I for observational studies. Of 593 records identified, three studies met the eligibility criteria: one randomized placebo-controlled trial and two observational studies. In the randomized trial of 58 patients, nabiximols reduced pain on movement by 0.95 points (*p* = 0.044), pain at rest by 1.04 points (*p* = 0.018), and improved sleep quality by 1.17 points (*p* = 0.027); DAS28 decreased by 0.76 points (*p* = 0.002), while no serious adverse events were reported. Observational studies suggested possible symptomatic benefit but were limited by heterogeneous cannabinoid exposure and serious to critical risk of bias. Current clinical evidence remains insufficient to establish the efficacy or long-term safety of cannabinoids for RA-related pain. Larger, rigorously designed trials using standardized cannabinoid formulations and pain-relevant outcomes are needed.

**Supplementary Information:**

The online version contains supplementary material available at https://doi.org/10.1007/s00296-026-06314-x.

## Introduction

Rheumatoid arthritis (RA) is a common systemic autoimmune disease associated with substantial morbidity, increased mortality and considerable socioeconomic burden [1]. Pain is considered one of its most prominent and disabling clinical manifestations, with up to 70% of patients identifying pain relief as their highest priority over other disease-related concerns [2]. Nearly all patients with early RA report pain, which is often a major reason for seeking medical care and a key contributor to psychological distress, sleep disturbances and functional impairment [2, 3].

Although major therapeutic advances and treat-to-target strategies have substantially improved disease control, a considerable proportion of patients continue to experience persistent pain [3]. Current evidence suggests that pain in RA is not solely driven by inflammation but also involves peripheral sensitization and altered central pain processing, which may promote the development of chronic refractory pain [3–6]. This multifactorial pain phenotype underscores the need for therapeutic approaches that extend beyond conventional analgesic strategies.

Against this background, cannabinoids have garnered increasing interest as potential modulators of RA-related pain, driven in part by expanding knowledge of the endocannabinoid system (ECS) and its role in nociception, neuroimmune signaling and inflammatory regulation [7]. The identification of cannabinoid receptors and endogenous ligands in joint tissues, together with preclinical findings suggesting that cannabidiol may attenuate inflammation and joint damage in experimental RA models, provides a biologically plausible rationale for targeting the ECS in this setting. Concurrently, increasing social acceptance and broader regulatory access to medical cannabinoids have made this issue highly relevant in clinical practice, as physicians are more frequently asked to advise patients who either self-medicate with cannabis products or consider cannabinoids as adjunctive treatment [8].

Despite growing interest in cannabinoids for chronic pain, the evidence specifically addressing pain in rheumatoid arthritis remains limited, heterogeneous, and methodologically diverse. A scoping review approach was therefore deemed appropriate to map the extent, nature, and characteristics of the available clinical evidence and to identify important knowledge gaps. The objective of this scoping review was to examine clinical studies of cannabinoid-related interventions or exposures in adults with rheumatoid arthritis, with particular focus on pain-related outcomes, safety, mechanistic relevance, and gaps that may inform future research and clinical decision-making.

### Pain in rheumatoid arthritis: beyond inflammation

Early initiation of treatment aiming at preventing irreversible joint damage is a fundamental principle of modern RA management [9]. Nevertheless, despite substantial advances in biologic and targeted therapies and the adoption of treat-to-target strategies, complete remission remains unattainable for a considerable proportion of patients and persistent pain remains a major unmet therapeutic need [10]. This challenge is particularly evident in difficult-to-treat RA (D2TRA), where persistent pain, functional impairment, treatment failure or intolerance, and psychosocial burden highlight the need for a broader, more holistic management approach [11, 12].

Traditionally, pain in RA has been conceptualized primarily as a consequence of inflammatory activity. However, although disease-modifying antirheumatic drugs (DMARDs) often reduce pain substantially, many patients continue to report clinically meaningful residual pain despite adequate control of inflammatory disease activity [13]. This discordance indicates that pain in RA cannot be explained by inflammation alone but rather reflects a complex interplay among peripheral tissue injury, inflammatory mediators, and altered nociceptive processing between both the peripheral and central nervous systems [2, 13, 14].

Key cytokines implicated in RA pathogenesis, including TNF-$\:{\upalpha\:}$, IL-1$\:{\upbeta\:}$, IL-6, and IL-17, appear to act directly on nociceptive neurons, increasing neuronal excitability and promoting peripheral sensitization [15]. At the same time, altered central pain modulation and transmission may amplify pain perception and contribute to hyperalgesia, allodynia and pain persistence even in the absence of overt synovitis. Central sensitization is therefore increasingly recognized as a clinically relevant mechanism in a subset of patients with RA, particularly when pain severity appears disproportionate to objective measures of inflammatory activity [13].

Beyond neurobiological mechanisms, psychosocial factors such as pain catastrophizing, anxiety and mood disorders also influence the perception and severity of pain. These factors have been associated with poorer long-term outcomes and reduced pain tolerance in patients with RA [4]. Clinically, this multidimensional nature of pain in RA suggests that escalation of immunomodulatory therapy alone may be insufficient when pain is driven, at least in part, by non-inflammatory mechanisms [6].

### The endocannabinoid system and pain modulation

Following the recognition that pain in RA cannot be explained solely by inflammatory activity, increasing attention has focused on endogenous regulatory systems involved in both nociception and immune homeostasis. Among these, the endocannabinoid system (ECS) is of particular interest as it modulates pain processing, inflammatory signaling and neuroimmune interactions [7, 16, 17]. At its core, the ECS consists primarily of cannabinoid receptors, endogenous lipid ligands and the enzymes responsible for their synthesis and degradation [7]. Its two best-characterized receptors, cannabinoid receptor type 1 (CB1) and cannabinoid receptor type 2 (CB2), are G protein-coupled receptors with distinct but partially overlapping distributions and functions [17, 18]. CB1 receptors are expressed predominantly within the central nervous system, where they regulate synaptic transmission and influence pain perception [7, 19, 20], whereas CB2 receptors are found mainly on immune cells and peripheral tissues, where they are more closely linked to immunomodulatory and anti-inflammatory effects [7, 17, 21, 22].

The principal endogenous cannabinoids, anandamide and 2-arachidonoylglycerol, are produced on demand [23] and act as retrograde signaling molecules that modulate neuronal excitability and neurotransmitter release [23–26]. Through these actions, the ECS contributes to the physiological modulation of nociceptive signaling at peripheral, spinal and supraspinal levels [17, 27–29]. Beyond pain processing, ECS-related signaling has been implicated in the regulation of cytokine production, immune cell activation and inflammatory pathways relevant to RA, including modulation of synovial fibroblast activity. Notably, CB2- associated pathways appear particularly relevant in RA, as they have been linked to attenuation of interleukin-1-driven inflammatory responses in RA synovial fibroblasts in experimental arthritis models [30–34].

Importantly, the biological effects of cannabinoids are unlikely to be mediated exclusively through CB1 and CB2 receptors. A growing body of evidence indicates that cannabinoids also interact with other molecular targets, including transient receptor potential channels such as TRPV1 and TRPA1, as well as nuclear receptors such as peroxisome proliferator-activated receptors (PPARs). Several of these pathways have been linked to reduced inflammatory signaling and improved pain-related outcomes in experimental RA models [7, 18, 35–37].

Collectively, these observations support the concept that the ECS may operate at the interface between pain and inflammation, making it an attractive therapeutic target in RA [37, 38]. Nevertheless, the extent to which this mechanistic rationale translates into meaningful clinical benefit remains uncertain [37], underscoring the need for a critical appraisal of the available clinical evidence.

## Methods

### Protocol and registration

This scoping review was reported in accordance with the Preferred Reporting Items for Systematic Reviews and Meta-Analyses extension for Scoping Reviews (PRISMA-ScR) [39]. In addition, the presentation and narrative synthesis of the evidence were informed by published recommendations for narrative biomedical reviews [40]. A formal review protocol was not prospectively registered. The review methods were predefined by the authors before study selection and data charting were undertaken.

### Eligibility criteria, information sources and search strategy

For this scoping review, a comprehensive literature search was conducted in PubMed/MEDLINE, Scopus, the Directory of Open Access Journals (DOAJ), and the Cochrane Central Register of Controlled Trials (CENTRAL) for studies published between January 1, 1990 and September 10, 2026. The search strategy combined free-text terms related to rheumatoid arthritis, cannabinoids, and pain, including “rheumatoid arthritis”, “RA”, “cannabinoids”, “cannabis”, “medical cannabis”, “cannabidiol”, “CBD”, “tetrahydrocannabinol”, “THC”, “nabiximols”, “Sativex”, “pain”, “analgesia” and “chronic pain”, with Boolean operators used to combine the main concepts. Search syntax was adapted to the requirements of each database. The complete database-specific search strategies are presented in Table 1.

**Table 1:** Search strategy

PubMed/MEDLINE	(“rheumatoid arthritis” OR “RA”) AND (“cannabinoid*” OR “cannabis” OR “THC” OR “tetrahydrocannabinol” OR “CBD” OR “cannabidiol” OR “nabiximols” OR “Sativex” OR “dronabinol” OR “nabilone”) AND (“pain” OR “analgesia” OR “analgesic” OR “pain management” OR “quality of life”)
Scopus	((“rheumatoid arthritis” OR RA) AND (“cannabinoid*” OR cannabis OR “medical cannabis” OR THC OR tetrahydrocannabinol OR CBD OR cannabidiol OR nabiximols OR Sativex OR dronabinol OR nabilone) AND (pain OR analgesia OR analgesic OR “pain management” OR “quality of life” OR function* OR disability))
Cochrane Central Register of Controlled Trials (CENTRAL)	(cannabis OR cannabinoid* OR cannabidiol OR CBD OR THC OR tetrahydrocannabinol OR nabiximols OR Sativex OR dronabinol OR nabilone) AND (rheumatoid arthritis OR RA)
Directory of Open Access Journals (DOAJ)	Separate searches combining the term “rheumatoid arthritis” with each cannabinoid-related term, including “cannabis”, “cannabinoid”, “cannabinoids”, “cannabidiol”, “CBD”, “THC”, “tetrahydrocannabinol”, “nabiximols”, “Sativex”, “dronabinol”, and “nabilone”

Eligible sources of evidence included full-text, peer-reviewed randomized controlled trials and observational studies evaluating cannabinoid-related interventions or exposures in adults with confirmed rheumatoid arthritis and reporting pain-related outcomes. Case reports, case series, reviews, editorials, conference abstracts, conference proceedings without a full-text peer-reviewed publication, preclinical studies, non-English publications, studies in mixed populations without separable rheumatoid arthritis data, and studies not reporting pain-related outcomes were excluded. The eligibility criteria were defined to capture clinically relevant human evidence on pain management in rheumatoid arthritis while excluding non- clinical or methodologically unsuitable sources.

### Selection of sources of evidence

**Fig. 1:**
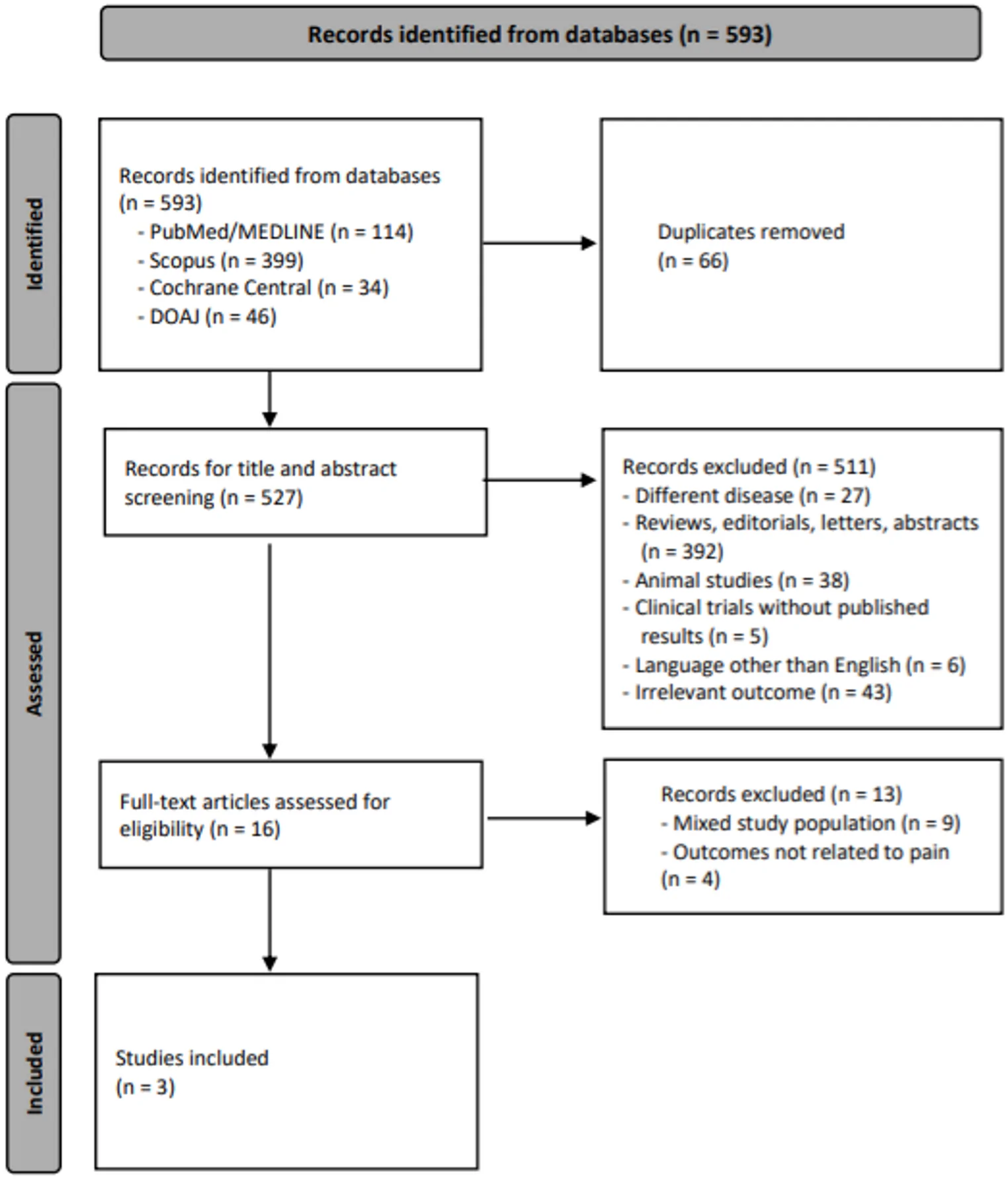
Flowchart summarizing the study selection process

Two authors (N.Z. and A.K.) independently screened the retrieved records by title and abstract, followed by independent full-text assessment of potentially eligible articles. Sources were included if they met the predefined eligibility criteria regarding population, exposure or intervention, and pain-related outcomes. Disagreements were resolved through discussion and, when necessary, by consultation of a third author (G.M.). Reasons for exclusion at the full-text stage were recorded and are summarized in the study flow diagram (Fig. 1).

### Data charting process and data items

Data were charted independently by two authors (N.Z. and A.K.) using a predefined data extraction approach developed for this scoping review. Extracted information was reviewed for consistency before synthesis. No additional contact with study investigators was undertaken for clarification or retrieval of missing data.

From each included source, the following data were sought: author, year, country, study design, study population, sample size, cannabinoid-related intervention or exposure, comparator when applicable, pain-related outcomes, additional clinical outcomes, adverse events, and key findings. Where relevant, mechanistic or disease-activity observations were also recorded to contextualize the clinical findings.

### Critical appraisal of individual sources of evidence

A critical appraisal was performed to provide additional context regarding the methodological limitations of the included clinical evidence. Risk of bias in the randomized trial was assessed using Cochrane RoB 2 [41], whereas the observational studies were appraised using ROBINS-I [42]. The results of this appraisal were used to contextualize the interpretation of findings and to strengthen the narrative synthesis of the evidence.

### AI disclosure

During manuscript preparation, artificial intelligence (AI) assistance (ChatGPT, OpenAI; GPT-5.6) was used from August 1 to September 11, 2026, to assist with language refinement and improvement of the clarity and organization of selected sections of the manuscript. All scientific content, interpretations, and conclusions were critically reviewed, verified, and approved by the authors, who take full responsibility for the final content of the manuscript.

## Results

The database search identified 593 records, including 114 from PubMed/MEDLINE, 399 from Scopus, 46 from Directory of Open Access Journals (DOAJ) and 34 from the Cochrane Central Register of Controlled Trials. Of these, three studies met the eligibility criteria and were included in the scoping review; one randomized placebo-controlled trial (Blake et al., 2006) [43] and two observational studies (Frane et al., 2022; Shrestha et al., 2025) [44, 45]. The study selection process is summarized in Fig. 1.

Given the small number of eligible studies and the marked heterogeneity in study design, populations, cannabinoid exposures, and outcome measures, a quantitative synthesis was not undertaken. The charted data were summarized descriptively and organized according to study characteristics, pain-related outcomes, safety findings, and major evidence gaps.

### Study characteristics

The only direct interventional study in RA was the double-blind, placebo-controlled, parallel-group trial by Blake et al. [43], which enrolled 58 patients with active RA who were already receiving DMARD therapy and evaluated an oromucosal spray delivering a fixed-ratio of tetrahydrocannabinol/cannabidiol (nabiximols, THC: CBD 1:1) over a five-week treatment period. The observational evidence was more indirect: Frane et al. [44] surveyed 428 adults with arthritis who had used CBD, including 142 patients with RA, whereas Shrestha et al. [45] analyzed 3,347,284 RA-related hospitalizations, 42,415 of which had a concomitant ICD-10 diagnosis of cannabis use. The main characteristics of the three included studies are summarized in Table 2.

**Table 2:** Summary of the included clinical studies evaluating cannabinoid-related interventions or exposures in patients with rheumatoid arthritis

Author (Year/ Country)	Design	Population	Cannabinoid exposure	Outcomes assessed	Main findings	Safety profile
Blake et al. (2006/UK) [43]	Randomized, double-blind, placebo-controlled, parallel-group trial	58 patients with active RA receiving DMARD treatment	Oromucosal nabiximols (THC: CBD 1:1) for 5 weeks	Pain on movement, pain at rest, sleep quality, morning stiffness, DAS28	Significant improvements in pain on movement (* -0.95* NRS points, *p* = 0.044), pain at rest (* -1.04*, *p* = 0.018), sleep quality (* -1.17*, *p* = 0.027) and DAS28 (* -0.76*, *p* = 0.002); no significant change in morning stiffness	Small sample and short follow-up. AEs mainly mild–moderate; no serious AEs or AE-related withdrawals in the active-treatment group
Frane et al. (2022/USA) [44]	Cross-sectional survey	428 adults with arthritis (including 142 patients with RA) who had used CBD	Self-directed CBD use; heterogeneous formulations, doses and duration	Patient-perceived pain, physical function, sleep quality, medication use, adverse effects	Overall arthritis cohort: 83% reported pain improvement (*p* = 0.072), 66% improvement in physical function (*p* = 0.013) and 66% improvement in sleep (*p* = 0.214); mean pain reduction 44% and 2.58 NRS points (*p* < 0.001). The majority reported reduction/discontinuation of other analgesics; RA-specific quantitative efficacy data were limited	41% reported ≥ 1 AE (dry mouth, somnolence, appetite changes, dry eyes, concentration problems, dizziness, headache and gastrointestinal symptoms), most of which were mild
Shrestha et al. (2025/USA) [45]	Observational NIS analysis	3,347,284 RA-related hospitalizations, including 42,415 with cannabis-use coding	Cannabis use identified through ICD-10 coding	Inpatient outcomes including mortality, chronic pain, depression, anxiety, opioid use, nicotine dependence and alcohol use	Cannabis use was associated with lower adjusted odds of mortality, chronic pain (OR: 0.45, *p* < 0.001), depression (OR: 0.55, *p* < 0.001) and anxiety (OR: 0.55, *p* < 0.001), but higher odds of opioid use (OR: 1.1, *p* < 0.001), nicotine dependence (OR: 1.35, *p* < 0.001) and alcohol use (OR: 1.35, *p* < 0.001); No significant difference in hospitalization duration (*p* = 0.1)	No direct safety assessment; administrative dataset not designed to evaluate cannabinoid-related adverse events

*AEs* Adverse Effects, *CBD* Cannabidiol, *THC* Tetrahydrocannabinol, *DMARD* Disease-Modifying Antirheumatic Drug, *DAS28* Disease Activity Score in 28 joints, *ICD-10* International Statistical Classification of Diseases – 10th Revision, *NIS* National Inpatient Sample, *NRS* Numerical Rating Scale, *OR* Odds Ratio, *RA* Rheumatoid Arthritis.

### Critical appraisal of individual sources of evidence

The Blake trial [43] was judged to be at low risk of bias across RoB 2 domains [41], although its small sample size and short follow-up limit generalizability rather than internal validity. By contrast, the study by Frane et al. [44] was judged to be at serious risk of bias, primarily because of concerns related to participant selection, self-reported exposure and outcomes, and the absence of a control group. Shrestha et al. [45] was judged to be at critical risk of bias under ROBINS-I [42] because of major concerns regarding confounding, participant selection, exposure classification, and outcome ascertainment. Detailed domain-level risk-of-bias assessments are provided in Supplementary Tables S1 and S2.

### Results of individual sources of evidence

In the randomized trial [43], nabiximols was associated with statistically significant improvements in pain on movement, which was reduced by a mean of 0.95 points on the Numerical Rating Scale (NRS) (*p* = 0.044). Pain at rest was reduced by 1.04 points (*p* = 0.018), and sleep quality was improved by 1.17 points (*p* = 0.027). Notably, the DAS28 score also improved, with a mean reduction of 0.76 points (*p* = 0.002), while morning stiffness did not change significantly.

In the cross-sectional survey by Frane et al. [44], CBD use was associated with patient-perceived improvement in pain, physical function and sleep quality across the entire arthritis cohort. Overall, 83% demonstrated improvement in pain, 66% in physical function, and 66% in sleep quality, while the cohort reported a 44% reduction in pain and a 2.58-point reduction on the numerical rating scale after CBD use (*p* < 0.001). The majority of respondents also reported reducing or discontinuing concomitant medications, including anti-inflammatory agents, acetaminophen and opioids, after CBD initiation. However, within the RA subgroup, responses were generally less pronounced than in osteoarthritis, and between-group differences reached statistical significance for physical function but were not consistently observed across all symptom domains. These findings are of clinical interest; however, their interpretation is inherently constrained by methodological limitations, including self-selection, recall bias, lack of a control group and substantial heterogeneity in CBD formulation, dose, frequency and duration. Also importantly, these quantitative symptom estimates were reported for the overall arthritis cohort and should not be interpreted as RA-specific treatment effects. RA-specific quantitative outcome data were limited and did not permit an independent estimate of treatment efficacy in the RA subgroup.

The National Inpatient Sample (NIS) analysis by Shrestha et al. [45] identified cannabis use in 1.27% of hospitalized patients with RA and found that cannabis use was associated with lower adjusted odds of mortality, chronic pain, depression and anxiety, but higher adjusted odds of opioid use, nicotine dependence and alcohol use. However, as exposure was defined through administrative ICD-10 coding and not by a standardized therapeutic cannabinoid intervention, these inpatient associations should be interpreted with caution when considering the efficacy of cannabinoid-based interventions in patients with RA.

Safety data were limited and derived primarily from short-term or observational evidence. In the Blake trial [43], there were no adverse-event-related withdrawals or serious adverse effects in the active-treatment group; however, the study included only 58 patients and had a treatment duration of five weeks. In the Frane survey [44] 41% of CBD users reported at least one adverse effect, most commonly dry mouth and somnolence. These findings are insufficient to establish the long-term safety or tolerability of cannabinoid-based interventions in RA.

Taken together, the included evidence was sparse and heterogeneous in design, exposure definition, clinical setting, and outcome assessment. Only one study provided direct randomized interventional evidence in patients with rheumatoid arthritis, whereas the remaining evidence was observational and methodologically constrained, providing substantially less direct evidence regarding therapeutic efficacy because of differences in population, exposure definition, and study design. Overall, the charted evidence may suggest potential symptomatic benefit of cannabinoid-related interventions or exposures for pain-related outcomes, but substantial uncertainty remains regarding efficacy, safety, and clinical applicability.

## Discussion

This scoping review demonstrates that the clinical evidence supporting cannabinoid use for pain management in RA remains remarkably limited, despite notable biological plausibility and increasing patient interest in cannabis-derived products. Of the 593 records identified, only three eligible clinical studies were included, comprising a single randomized controlled trial and two observational studies, an evidence base too small and heterogeneous to support definitive therapeutic conclusions.

The trial by Blake et al. [43] remains the only randomized controlled study directly evaluating a cannabinoid-based intervention in patients with RA and therefore represents the strongest available source of interventional evidence in this field. In that double-blind, placebo-controlled trial, 58 patients were randomized to oromucosal nabiximols or placebo for 5 weeks; active treatment was associated with statistically significant improvements in pain on movement, pain at rest, sleep quality and disease activity. However, the absolute reductions in pain were modest, at approximately 1 point on the NRS, and the clinical importance of these changes remains uncertain. Therefore, the statistically significant findings should not be interpreted as establishing a clinically meaningful analgesic benefit. Furthermore, the observed improvement in DAS28 should also be interpreted cautiously, as this composite score does not provide direct evidence of reduced inflammation and may be influenced by patient-reported components. In addition, the small sample size, short follow-up, evening-only dosing schedule and absence of longer-term outcomes substantially limit the strength and generalizability of these findings.

The two observational studies may provide some additional clinical context but do not materially strengthen causal inference and should not be interpreted as providing evidence equivalent to a randomized therapeutic trial. Frane et al. [44] reported perceived improvements in pain, sleep and physical function among respondents using CBD in the overall arthritis cohort; however, these findings derived from a self-selected online convenience sample, and the RA subgroup did not provide controlled efficacy evidence. The study is particularly susceptible to multiple sources of bias, including selection bias, recall bias, exposure heterogeneity and healthy-user bias, especially because recruitment occurred through advocacy networks and social media. Shrestha et al. [45], using NIS data, found that hospitalized RA patients coded as cannabis users had lower adjusted odds of mortality, depression, chronic pain and anxiety, but higher odds of opioid, nicotine and alcohol use. Yet the absence of formulation-specific exposure data, dose, duration, route of administration and timing of exposure make these associations non-causal and clinically difficult to translate.

From clinical perspective, the current evidence does not support routine use of cannabinoids for pain management in RA. Although the randomized trial demonstrated statistically significant reductions in pain, the absolute magnitude of improvement was modest, and the clinical relevance of this effect remains uncertain. The observational studies provide only indirect and methodologically limited evidence. Safety conclusions should also remain cautious, as the available studies were not designed to provide robust long-term safety data and the randomized evidence was limited to a small, five-week trial. Therefore, any potential symptomatic benefit should be weighed against the lack of robust efficacy data and the insufficient evidence regarding long-term safety.

Our findings align with previous systematic reviews, which have consistently concluded that the clinical evidence supporting cannabinoids in RA and related rheumatic diseases remains limited and methodologically weak [46–53]. Several additional clinical studies were identified through trial registries, including completed, suspended, and terminated studies. However, study registration or completion does not imply subsequent publication, and these studies cannot contribute to the current clinical evidence base in the absence of eligible peer-reviewed results. They are presented separately for transparency and were not included in the evidence synthesis (Table 3).

**Table 3:** Registered clinical studies without eligible full-text peer-reviewed results identified at the time of the search

Study ID/source	Study type	Intervention	Population	Registry status	Reason for non-inclusion
NCT04911127 [57]	Randomized, placebo-controlled, double-blind clinical trial	CBD	RA	Completed	No eligible full-text peer-reviewed publication identified
NCT05942911 [58]	Randomized, double-blind, placebo-controlled phase II clinical trial	Cannabinoid formulation (IHL-675 A).	RA	Terminated	No eligible full-text peer-reviewed publication identified
NCT07087938 [59]	Quantitative study	Cannabinoid (cannabichromene).	RA	Suspended	No eligible full-text peer-reviewed publication identified
NCT04269993 [60]	Counter-balanced, double-blind, crossover trial	Cannabinoids	RA, PsA	Terminated	Study funding was terminated; no eligible full-text peer-reviewed publication identified
ISRCTN29199098 [61]	Randomized, double-blind, placebo-controlled pilot study	Cannabinoids	RA	Completed	No eligible full-text peer-reviewed publication identified

*CBD* Cannabidiol, *PsA* Psoriatic Arthritis, *RA* Rheumatoid Arthritis

A central interpretive challenge arising from the limited available human data is that cannabinoids may confer potential benefits in pain, sleep and physical function; however, their anti-inflammatory effects are not clearly distinguished from nonspecific analgesia or from improvement in comorbid dimensions of chronic pain such as central sensitization, fibromyalgia or secondary osteoarthritis. This distinction is essential in rheumatoid arthritis, where residual pain often persists despite inflammatory control and may not track with conventional activity indices.

Previous publications in *Rheumatology International* have highlighted persistent pain, fatigue, impaired physical function, and reduced quality of life as important unmet needs in RA, including in patients with otherwise satisfactory inflammatory disease control. They have also emphasized the value of patient-reported outcomes and the multifactorial nature of residual pain [54–56]. The present scoping review adds to the existing literature by providing an updated and focused appraisal of cannabinoid-related clinical evidence specifically for pain management in rheumatoid arthritis. Unlike broader reviews addressing cannabinoids across rheumatic diseases or chronic pain conditions, this review distinguishes direct randomized interventional evidence from observational cannabis exposure, formally evaluates study-level risk of bias, and examines the clinical interpretability of reported pain outcomes. It also highlights the substantial discrepancy between biological plausibility and the very limited quality and quantity of available human evidence, thereby identifying specific methodological priorities for future RA-focused cannabinoid trials.

This scoping review has several limitations. First, the number of eligible clinical studies was very small, and the included evidence was highly heterogeneous in design, cannabinoid exposure, outcome definitions, and clinical setting. Second, only English-language publications were included, which may have limited capture of additional relevant evidence. Third, although critical appraisal was performed to contextualize the findings, most of the available evidence was observational and at serious or critical risk of bias, limiting confidence in the overall evidence landscape. Finally, additional studies were identified through clinical trial registries; however, because eligible peer-reviewed results were unavailable at the time of the search, they were not included in the evidence synthesis.

Future research should move beyond small exploratory studies and prioritize adequately powered, multicenter randomized trials using standardized cannabinoid formulations with longer treatment and follow-up periods. Core outcomes should include standardized measures of pain, disease activity, sleep, disability, mood, and safety, alongside careful recording of concomitant DMARD and analgesic use. Mechanistic enrichment would further strengthen the field, particularly through biomarker-based stratification, pharmacokinetic profiling and exploratory pharmacogenetic assessment of cannabinoid-related biomarkers. Pending the availability of such data, cannabinoids should remain an investigational approach in RA and cannot currently be recommended for routine evidence-based use.

## Conclusions

This scoping review highlights the marked paucity of high-quality clinical evidence supporting the use of cannabinoids for pain management in patients with RA. Of the three eligible studies identified, only one randomized controlled trial provided interventional data, demonstrating statistically significant improvements in pain and a reduction in DAS28, whereas the two observational studies were limited by substantial methodological weaknesses. Despite biological plausibility, the available clinical literature remains too sparse and heterogeneous to justify their routine evidence-based use in RA. Adequately powered randomized trials employing standardized cannabinoid formulations, rigorous outcome measures, and longer follow-up are needed to determine their efficacy and safety.

## Supplementary Information

Below is the link to the electronic supplementary material.

Supplementary Material 1
