# Supplementary material for: Cannabinoids for pain management in rheumatoid arthritis: a scoping review of clinical evidence and mechanisms
Source: Rheumatol Int. 2026 Oct 5;46(10):275. doi: 10.1007/s00296-026-06314-x (PMC13638674; doi:10.1007/s00296-026-06314-x)
Supplement: Supplementary file 1 [file 296_2026_6314_MOESM1_ESM.docx]

**Supplementary Table S1** Risk of bias assessment with Cochrane RoB 2[41]

| Domain  Study | D1 | D2 | D3 | D4 | D5 | Total |
| --- | --- | --- | --- | --- | --- | --- |
| Blake et al, 2006[43] | Low | Low | Low | Low | Low | **Low** |

***D1****: Bias arising from the randomization process;* ***D2****: Bias due to deviations from intended interventions;* ***D3****: Bias due to missing outcome data;* ***D4:*** *Bias in measurement of the outcome;* ***D5:*** *Bias in selection of the reported result*

**Supplementary Table S2** Risk of bias assessment with ROBINS-I[42]

| Domain  Study | D1 | D2 | D3 | D4 | D5 | D6 | D7 | Total |
| --- | --- | --- | --- | --- | --- | --- | --- | --- |
| Shrestha et al, 2025[45] | Critical | Serious | Critical | Moderate | Low | Moderate | Low | **Critical** |
| Frane et al, 2022[44] | Serious | Serious | Moderate | Moderate | Low | Moderate | Low | **Serious** |

***D1:*** *Confounding;* ***D2:*** *Selection bias;* ***D3:*** *Bias in classification of interventions;* ***D4:*** *Bias due to deviations from intended interventions;* ***D5:*** *Bias due to missing data;* ***D6:*** *Bias in measurement of outcomes;* ***D7:*** *Bias in selection of the reported result*
